# Supplementary material for: Rapid-charging aluminium-sulfur batteries operated at 85 °C with a quaternary molten salt electrolyte
Source: Nat Commun. 2024 Jan 18;15:596. doi: 10.1038/s41467-024-44691-8 (PMC10796388; doi:10.1038/s41467-024-44691-8)
Supplement: Supplementary file 1 — Supplementary Information [file 41467_2024_44691_MOESM1_ESM.pdf]

Supporting Information

Rapid-charging aluminum-sulfur batteries operated at 85 °C with a quaternary molten salt electrolyte

Jiashen Meng[1,2], Xufeng Hong[1], Zhitong Xiao[1], Linhan Xu[1], Lujun Zhu[1], Yongfeng Jia[1], Fang Liu[2], Liqiang Mai[2,], Quanquan Pang[1,*]*

1. Beijing Key Laboratory of Theory and Technology for Advanced Batteries Materials, School of Materials Science and Engineering, Peking University, Beijing, 100871, China

2. State Key Laboratory of Advanced Technology for Materials Synthesis and Processing, School of Materials Science and Engineering, Wuhan University of Technology, Wuhan 430070, P. R. China.

*Email: mlq518@whut.edu.cn; qqpang@pku.edu.cn

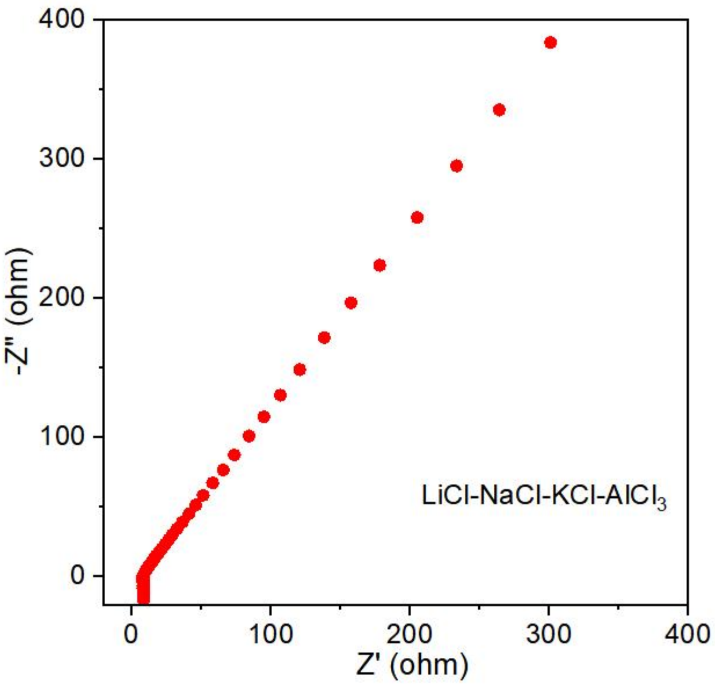

Supplementary Fig. 1 | The Nyquist plot of the Mo-Mo cell with LiCl-NaCl-KCl-$AlCl_3$ at 85 °C.

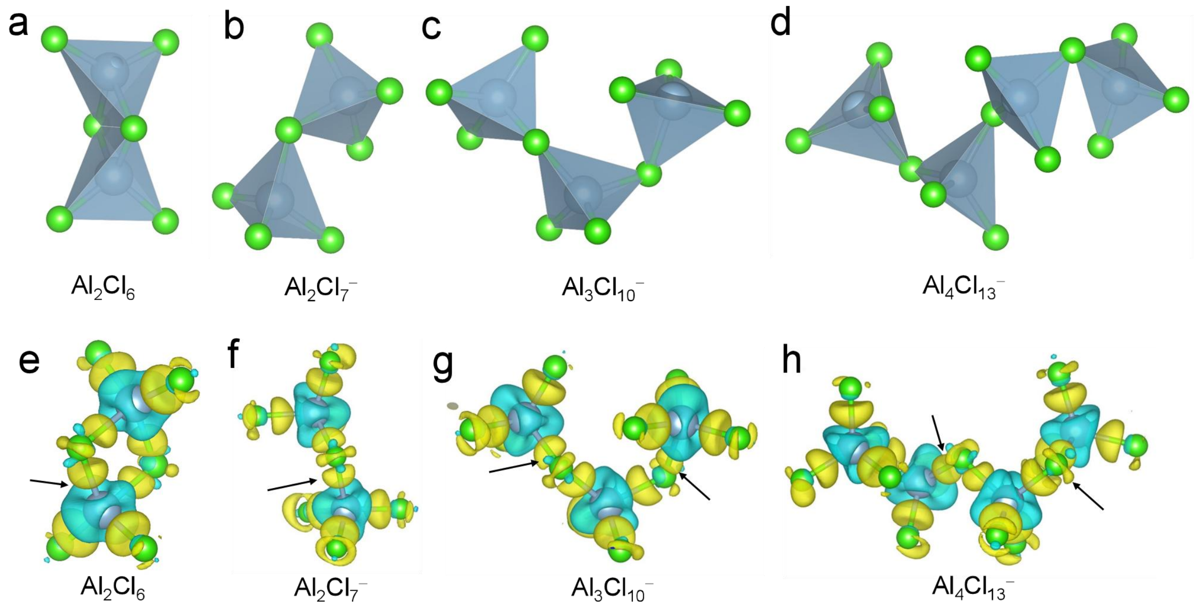

Supplementary Fig. 2 | Detailed atomic structures of the different Al-containing clusters as observed in the AIMD simulations. (a-d) Atomic structures of Al_2Cl_6, $Al_2Cl_7^-$, $Al_3Cl_{10}^-$ and $Al_4Cl_{13}^-$, in which green and gray balls represent Cl and Al atoms, respectively. (e-h) Deformation charge density of Al_2Cl_6, $Al_2Cl_7^-$, $Al_3Cl_{10}^-$ and $Al_4Cl_{13}^-$, in which blue and yellow regions indicate electron depletion and accumulation, respectively. The bridging Al-Cl bonds marked by arrows possess weaker deformation charge density than other Al-Cl bonds, indicating that the dissociation of the cluster may first occur on the bridging Al-Cl bonds.

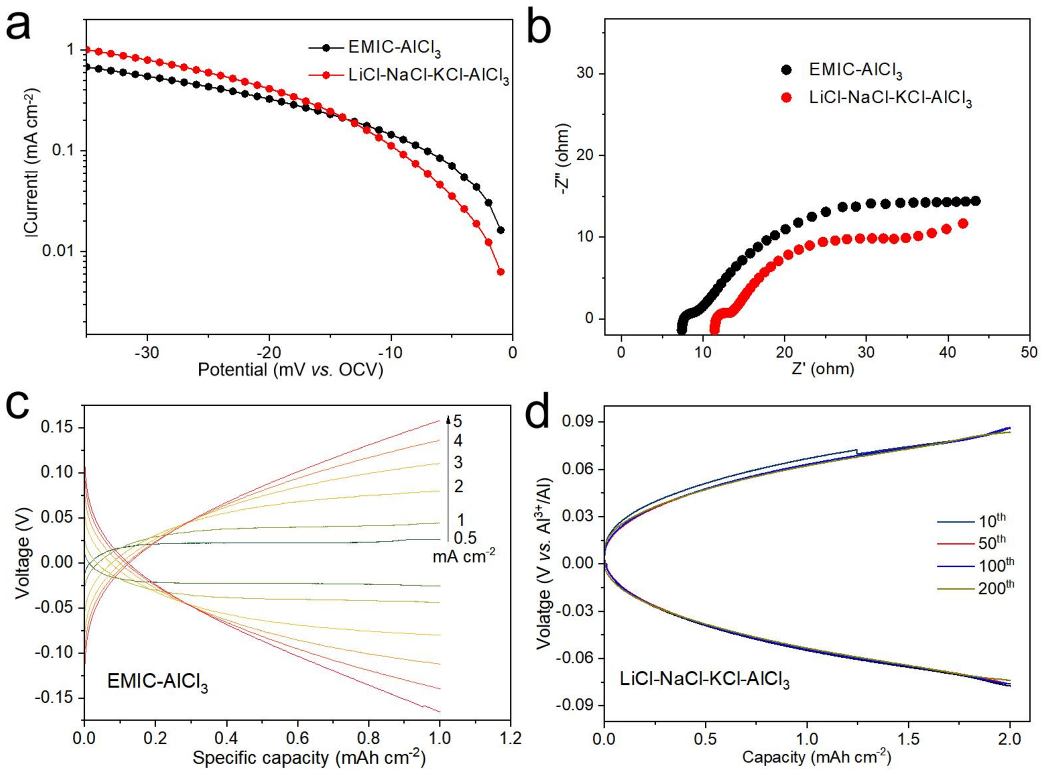

Supplementary Fig. 3 | The electrochemical evaluation of the quaternary alkali melt and ionic liquid electrolytes. (a, b) LSV curves and EIS spectra of the Al|Al symmetric cells at 85 °C using the quaternary alkali melt (LiCl-NaCl-KCl-$AlCl_3$) and the ionic liquid (EMIC-$AlCl_3$) electrolytes at the pristine state. (c) The voltage profiles of the Al|Al symmetric cell using the ionic liquid (EMIC-$AlCl_3$) electrolyte at different current densities. (d) The voltage profiles of the Al|Al symmetric cell using the quaternary alkali melt (LiCl-NaCl-KCl-$AlCl_3$) at a rate of 2 mA cm^{-2} with a constant capacity of 2 mA h cm^{-2}. The overlapping voltage profiles at different cycles indicate excellent Al plating/stripping stability in the quaternary alkali melt at 85 °C.

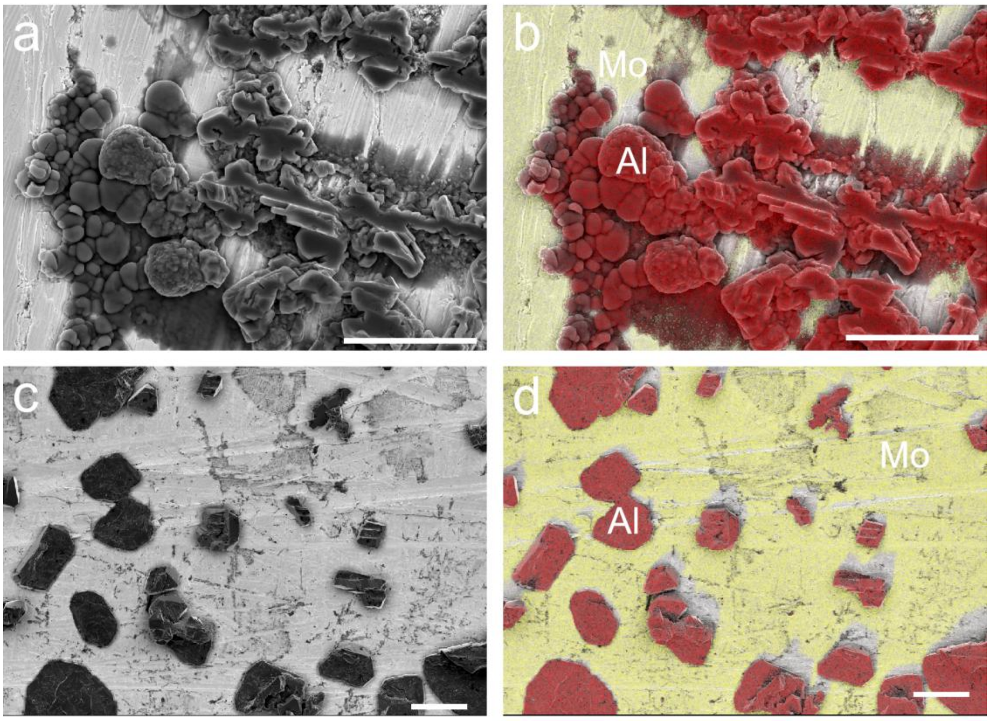

Supplementary Fig. 4 | Characterizations of the plated Al on Mo substrate in the Al-Mo cells using ionic liquid electrolyte and quaternary alkali chloroaluminate melt electrolyte. (a, b) SEM image and elemental mapping image of the plated Al on Mo substrate using ionic liquid electrolyte, scale bar: 30 µm. (c, d) SEM image and elemental mapping image of the plated Al on Mo substrate using quaternary melt electrolyte, scale bar: 50 µm.

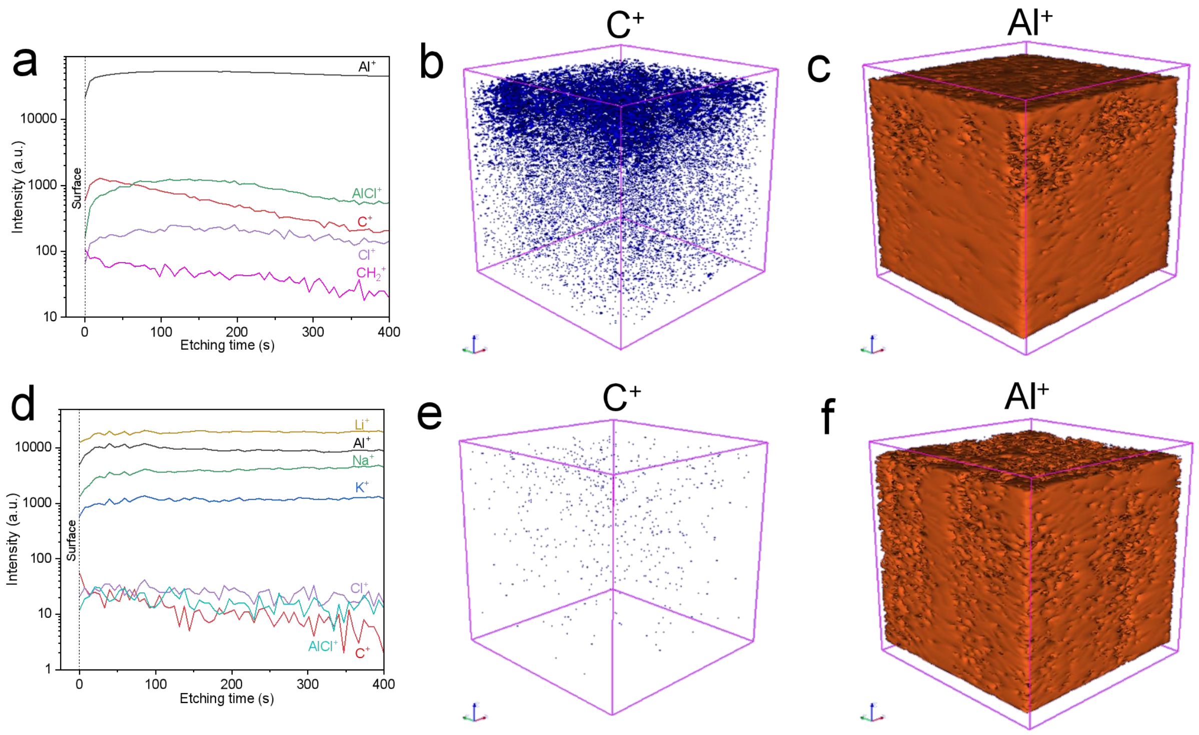

Supplementary Fig. 5 | TOF-SIMS of the Al anode surface after cycling in the ionic liquid and quaternary melt electrolytes. (a) Depth profiles of different secondary ions in the ionic liquid electrolyte. (b, c) 3D images of the sputtered volume corresponding to the depth profiles in the ionic liquid electrolyte. (d) Depth profiles of different secondary ions in the quaternary melt electrolyte. (e, f) 3D images of the sputtered volume corresponding to the depth profiles in the quaternary melt electrolyte.

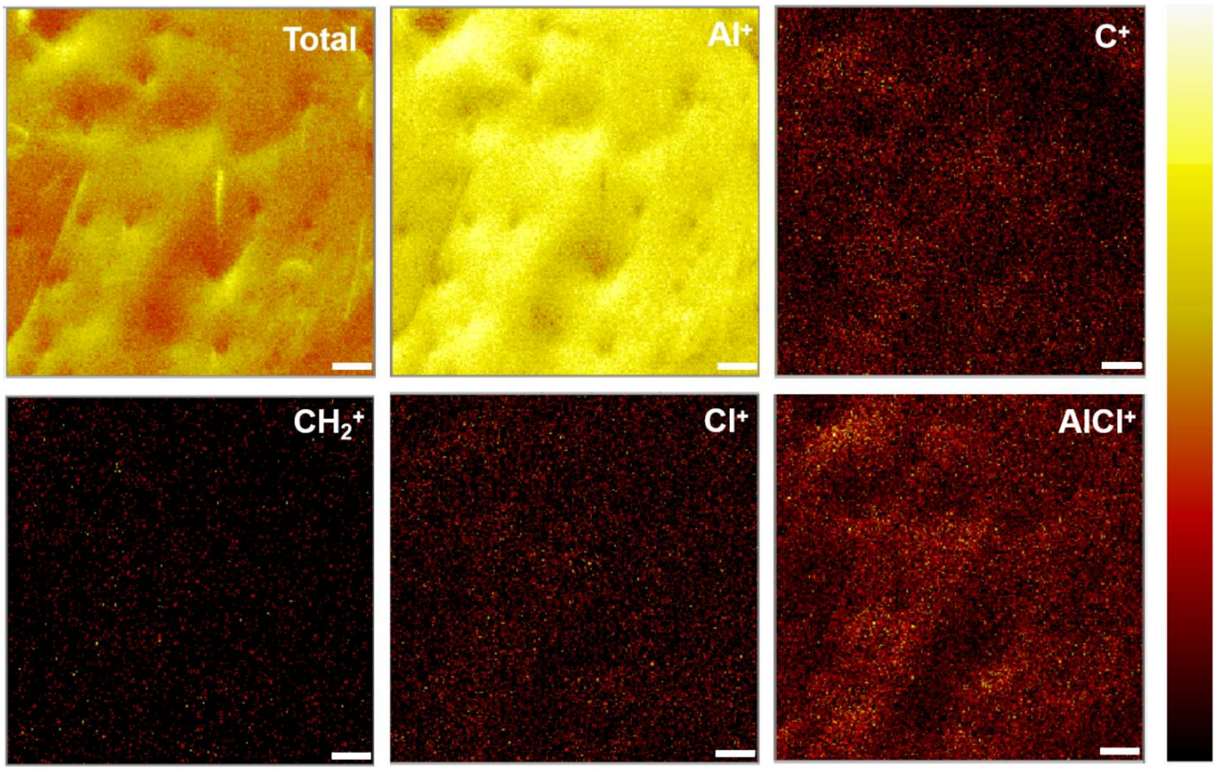

Supplementary Fig. 6 | TOF-SIMS 2D images of different secondary ions on the Al anode surface after cycling in the ionic liquid electrolyte. Scale bar: 10 μm.

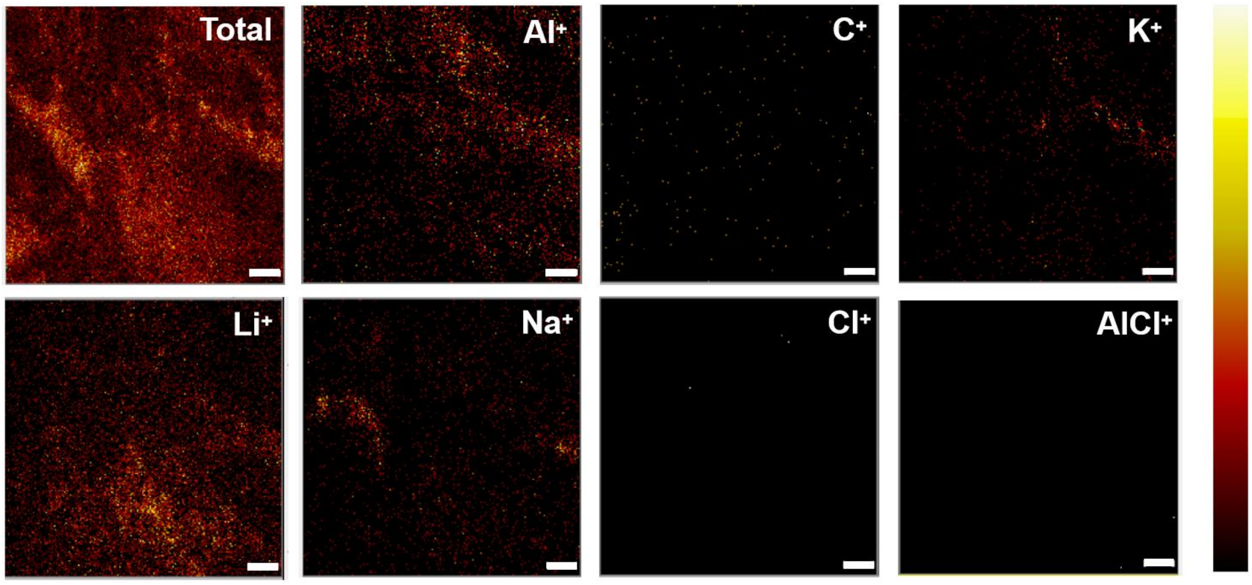

Supplementary Fig. 7 | TOF-SIMS 2D images of different secondary ions on the Al anode surface after cycling in the quaternary melt electrolyte. Scale bar: 10 μm.

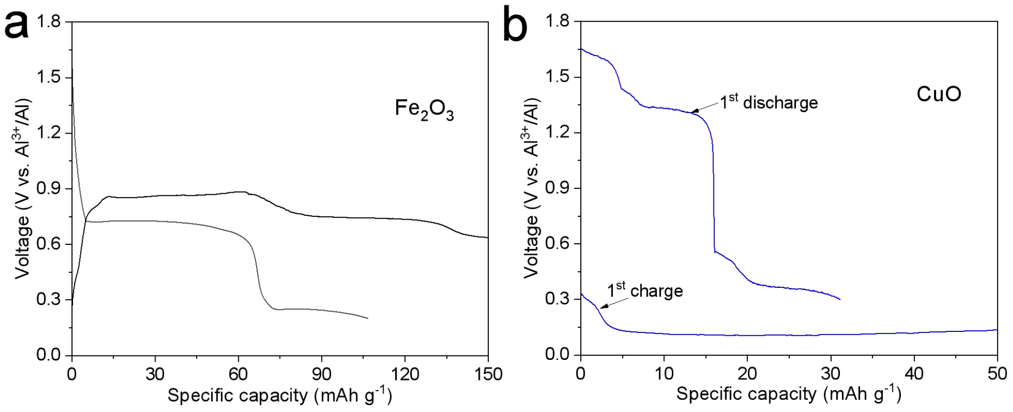

Supplementary Fig. 8 | Electrochemical behaviors of various metal oxides in the quaternary alkali melt electrolyte. The initial cycle voltage profiles of the (a) Al-Fe_2O_3 and (b) Al-CuO batteries using the quaternary alkali melt electrolyte at 20 mA g^{-1} and 85 °C. It is clear that although the two materials show sensible discharge behaviors in the molten salt electrolyte during the first discharge, they experience poor rechargeability over the first charge. In fact, upon observation of such types of materials in the molten salt electrolyte, they show very high reactivity with the electrolyte, as well as change of color of the electrolyte solution. This indicates that some metal oxides are not compatible with the reactive molten salt electrolytes and are thus not appropriate for use of catalytic sulfur host materials. This is likely to the breaking of metal-oxygen bonds in the chloroaluminate molten salts.

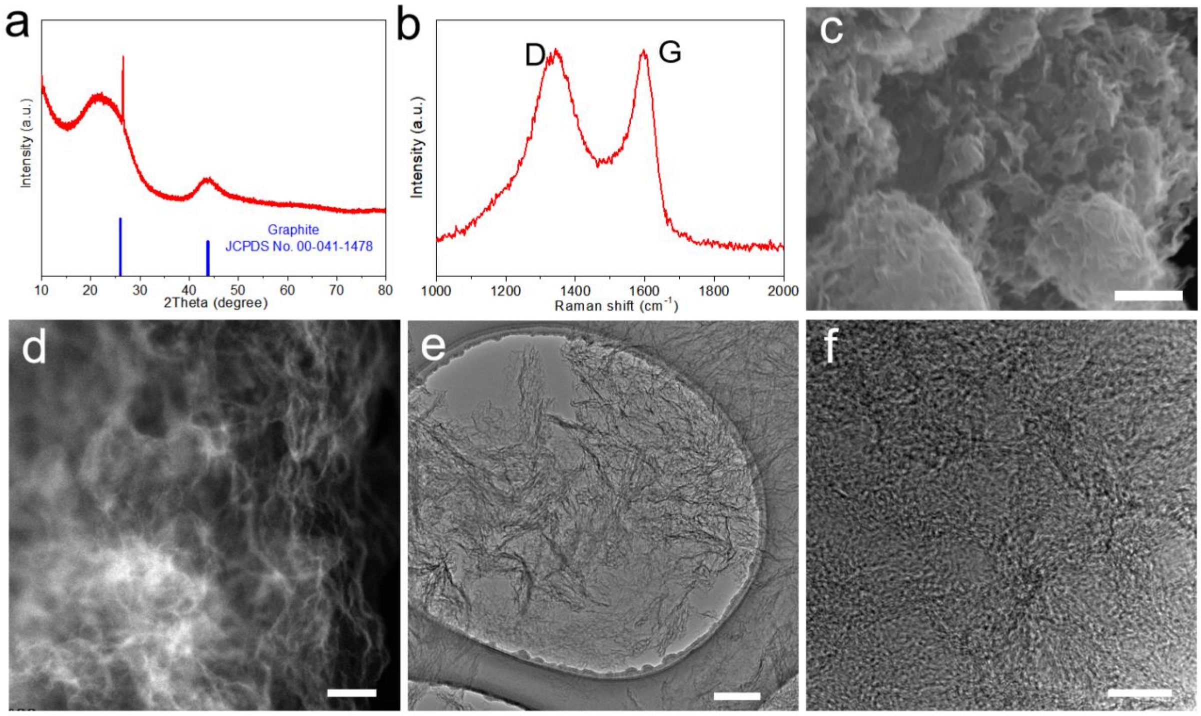

Supplementary Fig. 9 | Structural characterizations of the synthesized NCF material. (a) The XRD pattern, (b) Raman spectrum, (c) SEM image, (d) HADDF-STEM image and (e, f) TEM images of the NCF material. (c) Scale bar: 1 μm, (d) Scale bar: 50 nm, (e) Scale bar: 200 nm, (f) Scale bar: 10 μm. The XRD pattern shows a typical diffraction peak located at ~26°, corresponding to the (002) plane of graphite. Raman spectrum exhibits two obvious D and G bands, indicating partial graphitization and rich defects in NCF. The electron microscopy images show that NCF presents an interconnected hollow framework with thin shells of a few nanometers.

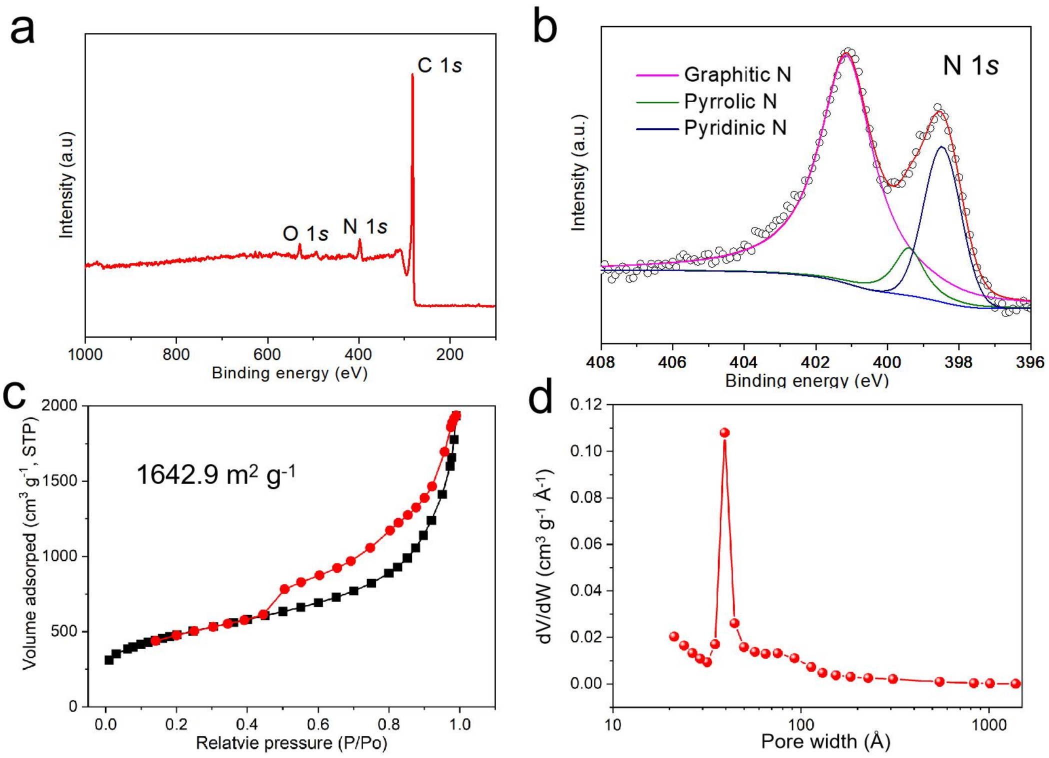

Supplementary Fig. 10 | Chemical and porosity characterizations of the NCF material. (a) XPS full spectrum, (b) high-resolution XPS N *1s* spectrum, (c) the BET absorption isotherm curve, and (d) pore width distribution of the NCF material. The XPS full spectrum shows the presence of C, N and O, and on basis of the high-resolution N *1s* XPS spectrum, three types of nitrogen species are identified, including graphitic N, pyrrolic N and pyridinic N, which may act as catalytic sites to improve the sulfur conversion kinetics. From the nitrogen adsorption-desorption isotherm, the NCF is calculated to exhibit a high surface area of 1642.9 m^2 g^{-1}.

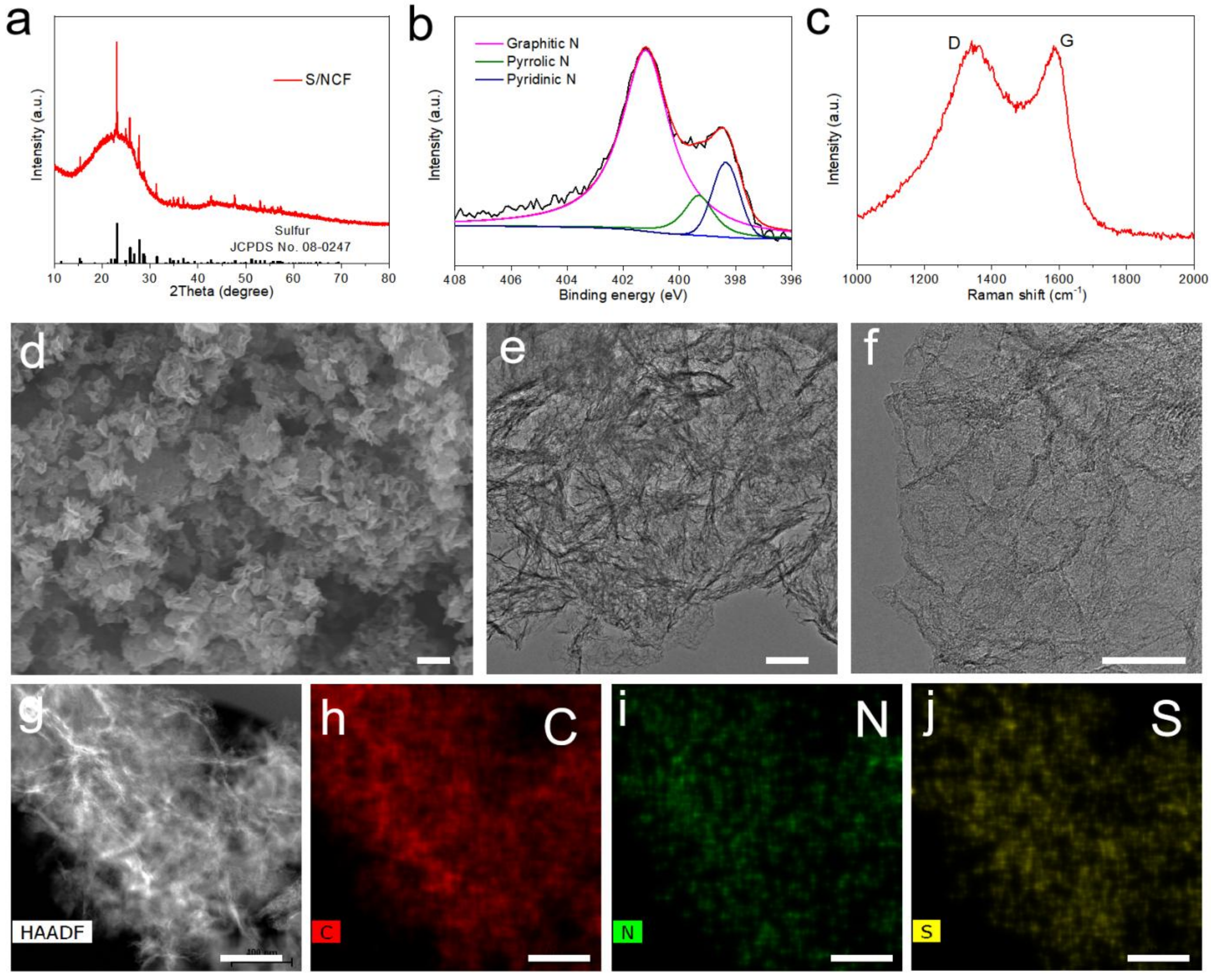

Supplementary Fig. 11 | Structure characterizations of the S/NCF composite. (a) XRD pattern, (b) High-resolution N 1*s* XPS spectrum, (c) Raman spectrum, (d) SEM image, (e, f) TEM images, (g-j) HADDF STEM image and the corresponding elemental mapping images of the S/NCF composite. (d) Scale bar: 1 μm, (e) Scale bar: 200 nm, (f) Scale bar: 50 nm, (g-j) Scale bar: 400 nm. The XRD pattern of the resulting S/NCF shows that all diffraction peaks are indexed to sulfur (JCPDS no. 08-0247). The high-resolution N 1*s* XPS spectrum and the Raman spectrum show that the nitrogen groups and the degree of graphitization remains after sulfur infiltration. The SEM and TEM images show the absence of large sulfur aggregate, confirming uniform distribution of the sulfur species on the NCF. Furthermore, the HAADF-STEM image and the corresponding mapping images display uniform distribution of C, N, and S elements in the S/NCF composite.

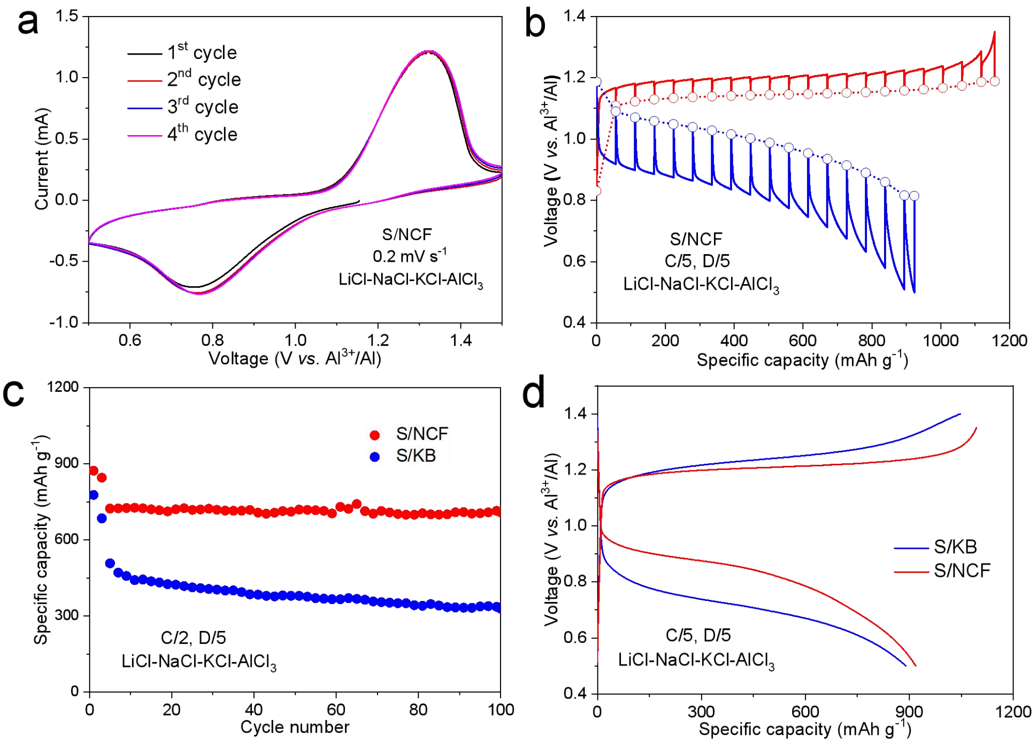

Supplementary Fig. 12 | Electrochemical performance of the Al-S battery using quaternary alkali chloroaluminate melt electrolyte at 85 °C. (a) CV curves of the Al-S battery using S/NCF cathode and quaternary alkali chloroaluminate melt at a scan rate of 0.2 mV s^{-1}. (b) The GITT voltage profile of the molten salt Al-S battery using S/NCF cathode and quaternary alkali chloroaluminate melt at a discharging/charging rate of C/5. (c) Cycling performance of the Al-S battery using S/NCF and S/KB cathodes at a charging rate of C/2 and a discharging rate of D/5. (d) The voltage profiles of the Al-S battery using S/KB cathode and S/NCF cathode at a discharging/charging rate of C/5.

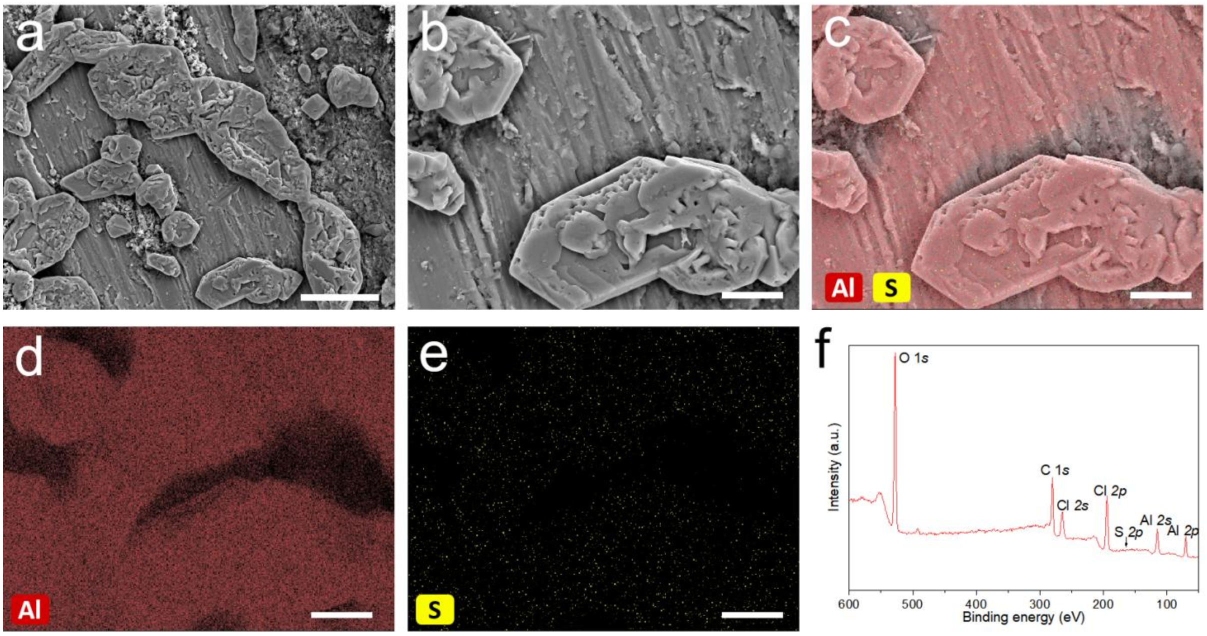

Supplementary Fig. 13 | Characterizations of the Al anode in molten salt Al-S battery after cycling at C/5 and 85 °C. (a) SEM image, scale bar: 40 μm. (b-e) SEM image and corresponding elemental mappings, scale bar: 10 μm. (f) XPS full spectrum. The SEM image shows compact micron-sized crystals on the Al surface. SEM image and the corresponding elemental mappings indicate that the Al anode is sulfur-free (at least to the limits of detection of energy-dispersive X-ray spectroscopy). In addition, high-resolution XPS measurement was performed to explore the surface information of the Al anode. There are no sulfur signals in the XPS full spectrum.

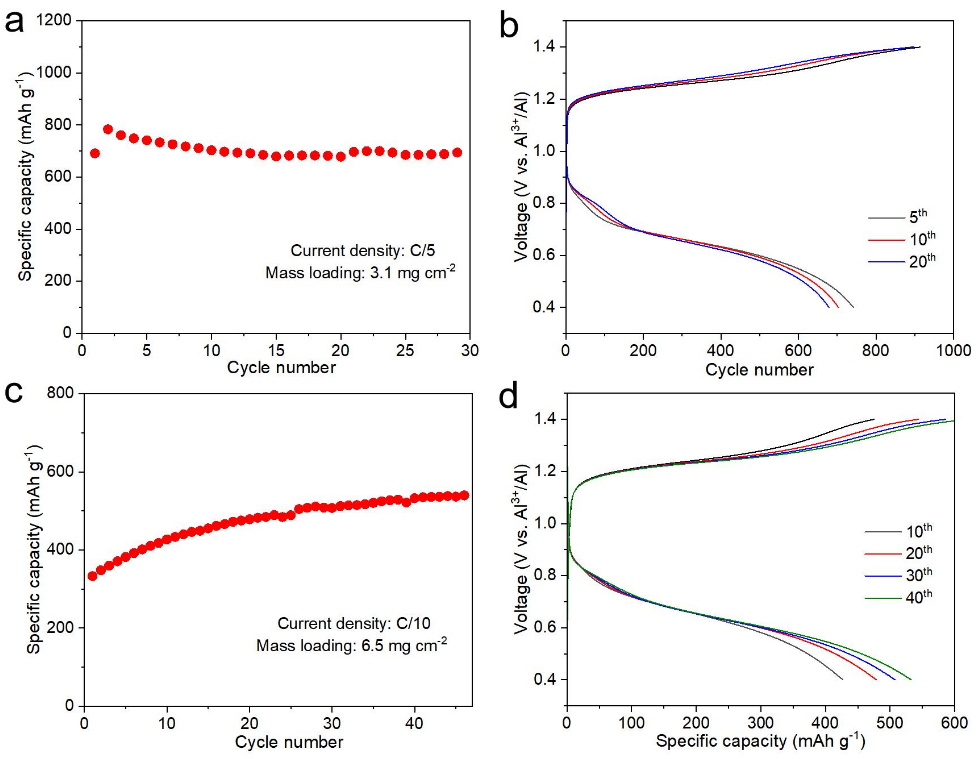

Supplementary Fig. 14 | Electrochemical performance of the Al-S battery using the quaternary molten salt electrolyte at high sulfur loading. (a, b) Cycling performance and voltage profiles of the S electrode with a mass loading of 3.1 mg cm^{-2} at C/5 and 85 °C. (c, d) Cycling performance and voltage profiles of the S electrode with a mass loading of 6.5 mg cm^{-2} at C/10 and 85 °C.

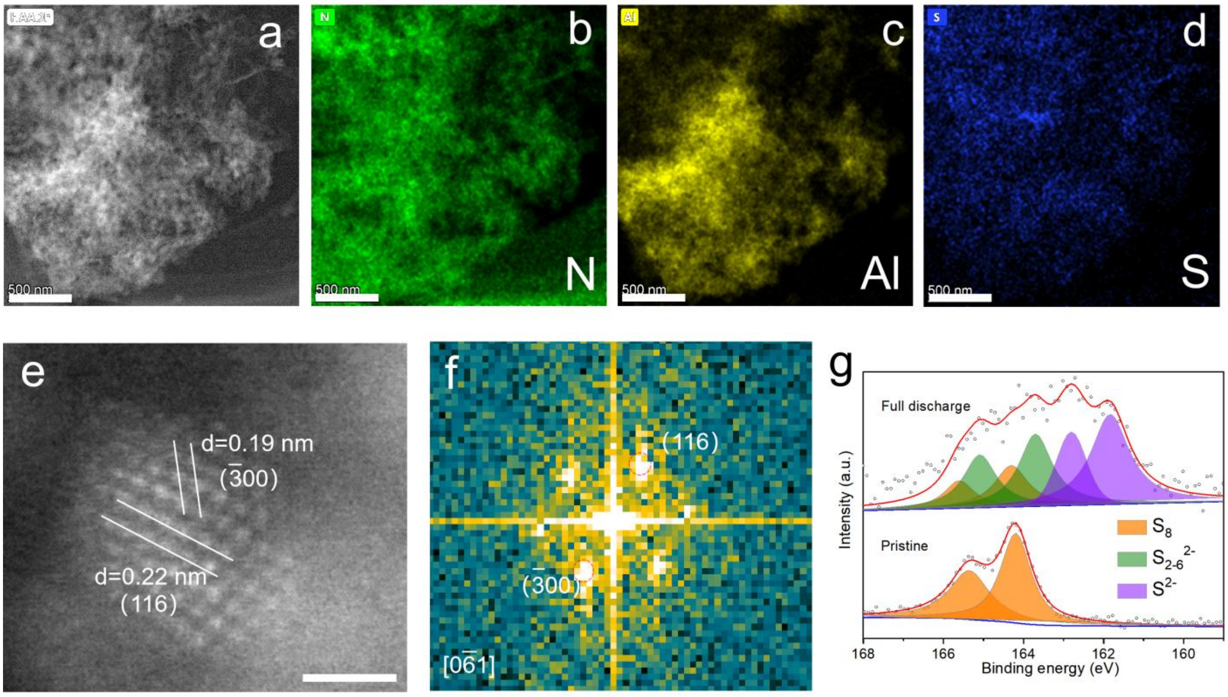

Supplementary Fig. 15 | Structure characterizations of the S/NCF cathode after full discharge in the molten salt Al-S battery. (a-d) HAADF-STEM image and corresponding elemental mapping images, (e-g) HRTEM image and FFT pattern, and the S *2p* XPS spectra of the S/NCF composite cathode after full discharge, (a-d) Scale bar: 500 nm, (e) Scale bar: 1 nm. In the panel g, the dotted line and the red line represents the raw data and fitted overall data, and the painted areas represent the fitted component spectra, where at least six spectra must be included to fit the spectra.

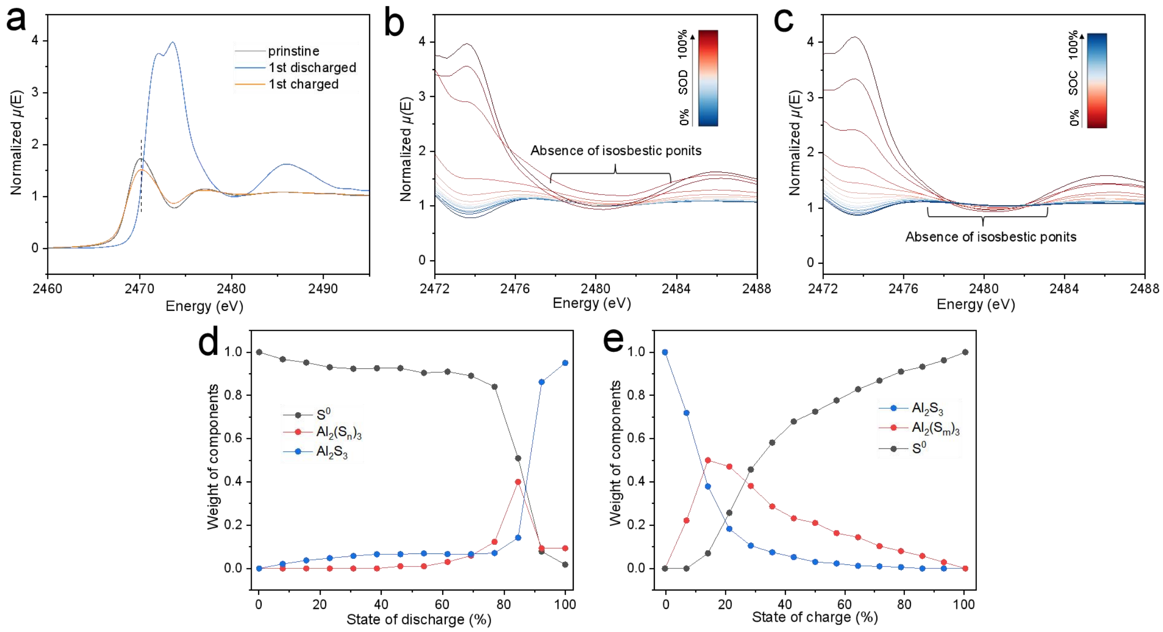

Supplementary Fig. 16 | Analyses of the S K-edge XANES spectra to reveal the reaction pathways of the molten salt Al-S battery. (a) Some representative XANES spectra of the sulfur cathode at the pristine, 1st discharged, and 1st charged states. The intensity difference of the XANES spectra at the pristine and 1st charged states can be attributed to the different sulfur particle size in the sulfur cathode, thus leading to different degree of sulfur self-absorption effect. (b, c) The magnified XANES spectra of the sulfur cathode during discharge and charge in representative regions that show the absence of isosbestic points, which means that not all spectra have a single comment interconnection point. (d, e) The weight of each component as quantified by linear combination fitting as a function of the SOD during discharge (d) and as a function of the SOC during charge.

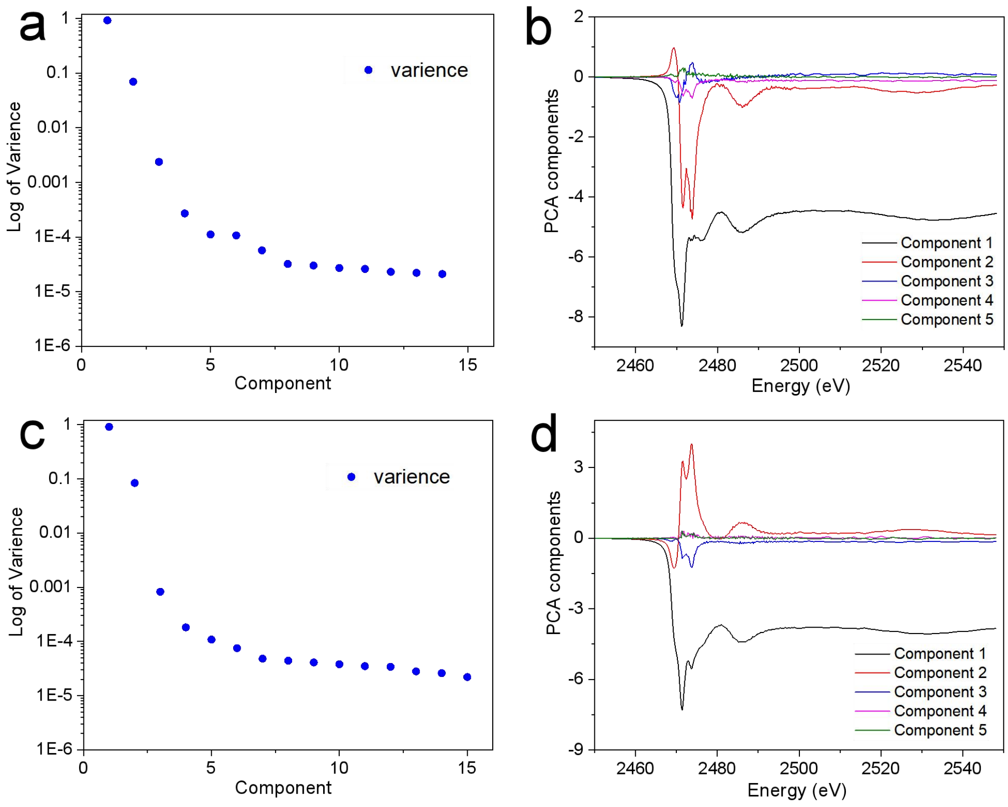

Supplementary Fig. 17 | The principal component analyses for XANES spectra during cycling. (a) The plots of logarithmic variance of each component for the PCA during discharging. (b) The plots of first five PCA-derived components during discharging. (c) The plots of logarithmic variance of each component for the PCA during charging. (d) The plots of first five PCA-derived components during charging.

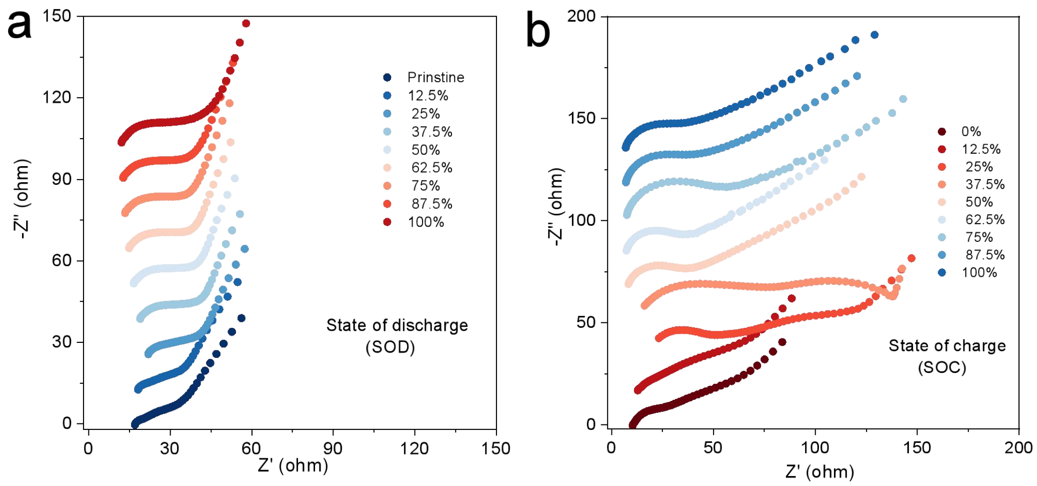

Supplementary Fig. 18 | *Operando* EIS studies on the Al-S battery using the quaternary melt electrolyte during the first cycle. *Operando* time-lapse EIS profiles of the S/NCF cathode at different (a) states of discharge (SOD) and (b) states of charge (SOC). The SOD and SOC are calculated based on the capacity over the total capacity.

Due to the variation of the shape of the Nyquist plots (and thus the equivalent circuits)on charge, we did not attempt to fit the spectra with equivalent circuits, but instead carried out qualitative analyses. First, during discharge, the charge transfer resistance (R_{ct}, as qualitatively represented by the magnitude of the semi-circle) vary little; however, during charge (SOC:25%-37.5%), a second semicircle at medium frequencies appears, indicating the formation of a new intermediate phase, which is believed to be crucial for fast-charging of Al-S battery. Second, the solution resistance (represented by the intercept with the Z' axis) shows slight increase when discharge or charge initiates, and then return to almost the original values at the end of discharge or charge; this is strong evidence that the aluminium polysulfides are formed and partially soluble in the molten salt electrolyte, which is central to the fast rate capability of the cell.

Supplementary Table 1 | Summary on the melting point of chloride salts with varied ratios.

**Molten salts**	**Salt components**	**Molar ratios**	**Melting point (^{o}C)**
Single	LiCl	1	605
	NaCl	1	801
	KCl	1	858
	$AlCl_3$	1	190
Binary	LiCl/NaCl	72/28	549
	KCl/NaCl	50/50	652
	KCl/LiCl	42/58	354
	$AlCl_3$/LiCl	58/42	110
	$AlCl_3$/NaCl	61/39	102
	$AlCl_3$/KCl	66/34	128
Ternary	$AlCl_3$/NaCl/KCl	61/26/13	~100
	$AlCl_3$/LiCl/KCl	59/29/12	~95
Quaternary	$AlCl_3$/LiCl/NaCl/KCl	120/42/43/15	~80

Supplementary Table 2 | A comparison of our work and previous reports on the electrochemical performance of Al-S batteries.

Cathode structures	Electrolyte components	Operation temperature (°C)	Current density	Cycle number	Residual capacity ($mAh\ g^{-1}$)	Capacity Retention (%)	References
**S/NCF**	**LiCl-NaCl-KCl-$AlCl_3$**	**85**	**0.2C**<br>**1C**	**200**<br>**1400**	**869**<br>**542**	**93.4%**<br>**85.4%**	**Our work**
S@N-doped hierarchical porous carbon	Acetamide-$AlCl_3$	25	0.2 $A\ g^{-1}$<br>1 $A\ g^{-1}$	50<br>700	1027<br>405	57.2%<br>45%	[1]
S@$Co^{II,III}$ decorated carbon matrix	EMIC-$AlCl_3$	25	1 $A\ g^{-1}$	200	500	33.3%	[2]
S@CMK-3	NBMPBr-$AlCl_3$	25	0.25 $A\ g^{-1}$	20	420	30.2%	[3]
S@CMK-3	Acetamide-$AlCl_3$	25	0.1 $A\ g^{-1}$	60	500	25%	[4]
S@carbon nanofiber paper	EMIC-$AlCl_3$-$LiCF_3SO_3$	25	C/20	50	600	60%	[5]
S@multiwalled carbon nanotube	Urea-$AlCl_3$	25	1 $A\ g^{-1}$	100	510	68%	[6]
S@activated carbon cloth	EMIC-$AlCl_3$	25	0.05 $A\ g^{-1}$	20	1000	75.8%	[7]
S@$Ti_3C_2T_x$	EMIC-$AlCl_3$	25	0.3 $A\ g^{-1}$	280	415	84.9%	[8]
S@Co/N co-doped graphene	EMIC-$AlCl_3$@MOF	25	0.05 $A\ g^{-1}$	300	640	78%	[9]
S@TiN/graphene	EMIC-$AlCl_3$	25	0.1 $A\ g^{-1}$	200	506	51%	[10]
S@Graphene	NaCl-KCl-$AlCl_3$	110	50C	200	345	86%	[11]

Note that the capacity retention is based on the ratio of the residual capacity to the maximum capacity in the initial cycles. Here 1 C is the current density of 1675 $mA\ g^{-1}$.

References

1 Zhang, D. *et al.* Highly reversible aluminium–sulfur batteries obtained through effective sulfur confinement with hierarchical porous carbon. *J. Mater. Chem. A* **9**, 8966-8974 (2021).

2 Guo, Y. *et al.* Rechargeable aluminium-sulfur battery with improved electrochemical performance by cobalt-containing electrocatalyst. *Angew. Chem. Int. Ed.* **59**, 22963-22967 (2020).

3 Yang, H. *et al.* An aluminum-sulfur battery with a fast kinetic response. *Angew. Chem. Int. Ed.* **57**, 1898-1902 (2018).

4 Chu, W. *et al.* A low-cost deep eutectic solvent electrolyte for rechargeable aluminum-sulfur battery. *Energy Storage Mater.* **22**, 418-423 (2019).

5 Yu, X., Boyer, M. J., Hwang, G. S. & Manthiram, A. Room-temperature aluminum-sulfur batteries with a lithium-ion-mediated ionic liquid electrolyte. *Chem* **4**, 586-598 (2018).

6 Bian, Y. *et al.* Using an $AlCl_3$/urea ionic liquid analog electrolyte for improving the lifetime of aluminum-sulfur batteries. *ChemElectroChem* **5**, 3607-3611 (2018).

7 Gao, T. *et al.* A rechargeable Al/S battery with an ionic-liquid electrolyte. *Angew. Chem. Int. Ed.* **55**, 9898-9901 (2016).

8 Zheng, X., Wang, Z., Li, J. & Wei, L. Binder-free S@$Ti_3C_2T_x$ sandwich structure film as a high-capacity cathode for a stable aluminum-sulfur battery. *Sci. China Mater.* **65**, 1463-1475 (2022).

9 Huang, Z. *et al.* Electrocatalysis for continuous multi-step reactions in quasi-solid-state electrolytes towards high-energy and long-life aluminum-sulfur batteries. *Angew. Chem. Int. Ed.* **61**, e202202696 (2022).

10 Ai, Y. *et al.* Bifunctional TiN@N-doped-graphene catalyst based high sulfur content cathode for reversible aluminum-sulfur batteries. *Energy Storage Mater.* **48**, 297-305 (2022).

11 Pang, Q. *et al.* Fast-charging aluminium-chalcogen batteries resistant to dendritic shorting. *Nature* **608**, 704-711 (2022).
