# Supplementary material for: Rapid-charging aluminium-sulfur batteries operated at 85 °C with a quaternary molten salt electrolyte
Source: Nat Commun. 2024 Jan 18;15:596. doi: 10.1038/s41467-024-44691-8 (PMC10796388; doi:10.1038/s41467-024-44691-8)
Supplement: Supplementary file 3 — Description of Additional Supplementary Files [file 41467_2024_44691_MOESM3_ESM.pdf]

Description of Additional Supplementary Files

Supplementary Movie 1 Ab initio molecular dynamics simulations of the EMIC-$AlCl_3$ ionic liquid electrolyte.

Supplementary Movie 2 Ab initio molecular dynamics simulations of the LiCl-NaCl-KCl-$AlCl_3$ molten salt electrolyte.
